# Optimal Timing of Oral Anticoagulant Therapy Initiation Post-acute Ischemic Stroke: A Narrative Review

**DOI:** 10.7759/cureus.93275

**Published:** 2025-09-26

**Authors:** Nicholas Aderinto, Lama H Aljamea, Faisal H Aljamea

**Affiliations:** 1 Medicine, Ladoke Akintola University of Technology, Ogbomoso, NGA; 2 College of Medicine, King Faisal University, Al-Ahsa, SAU; 3 Medicine and Surgery, Vision College, Riyadh, Riyadh, SAU

**Keywords:** acute ischaemic stroke, atrial fibrillation, direct oral anticoagulants (doacs), oral anticoagulants, therapy initiation

## Abstract

Initiation of oral anticoagulation therapy after acute ischemic stroke requires the best trade-off between prevention of early recurrent embolic events and hemorrhagic transformation avoidance. While guidelines typically recommend starting therapy between 4 and 14 days, current evidence suggests that timing should be tailored to stroke severity, imaging findings, and individualized patient characteristics. This narrative review summarizes evidence from guidelines and clinical trials/observational studies published between 2013 and 2025, identified through PubMed, Web of Science, and Google Scholar. Summary of recent trials, including ELAN and TIMING, demonstrates that early initiation, i.e., within 48 hours to five days after minor or moderate strokes, is generally safe and associated with improved functional outcome and reduced mortality, without the risk of increased bleeding. Delayed initiation, however, is typically associated with augmented embolic events and does not provide an additional safety benefit. In general, the literature supports a patient-centered approach where early treatment is desirable for most cases, but larger infarcts, hemorrhage signs, or previous surgical procedures might call for a more cautious delay to establish stability.

## Introduction and background

The urgency stems from the necessity to prevent recurrent thromboembolic events, while the risk of hemorrhagic transformation (HT) in the infarcted tissue complicates early intervention [1]. This balance necessitates a nuanced approach, carefully weighing potential benefits against the risks of exacerbating intracranial bleeding [2]. Favorable outcomes after an ischemic stroke depend on the quick administration of therapies like intravenous thrombolysis and mechanical thrombectomy [3]. These interventions aim to restore cerebral blood flow, but subsequent anticoagulation requires careful timing [1,4]. Guidelines suggest initiating anticoagulation between 4 and 14 days post-cardioembolic stroke, but these recommendations may not account for critical factors like infarct size or HT [5]. Such factors require consideration alongside logistical obstacles and high costs when deciding on the optimal intervention procedure [1,6]. Therefore, current guidelines may not address situations with limited scientific evidence, further complicating clinical decision-making [7]. The expansion of therapeutic windows reflects the evolving understanding and management of acute ischemic stroke (AIS), prompting a re-evaluation of existing protocols [8]. The importance of early detection and triage is increasingly emphasized, especially since a notable percentage of strokes occur in hospitalized patients. This balance is further challenged by the disparities between official drug labels, guidelines, and expert recommendations, necessitating a critical appraisal of randomized controlled trials (RCTs) and cohort studies [9]. While oral anticoagulants (OACs) are effective for various conditions, they also increase the risk of bleeding events, necessitating close monitoring [10]. A comprehensive approach to stroke prevention, including lifestyle adjustments and targeted pharmacological interventions, could significantly reduce the risk of recurrent events [11]. This necessitates continuous refinement of treatment strategies to improve stroke outcomes and reduce disability [12].

Background and rationale

What Is the Underlying Pathophysiological Rationale Supporting Anticoagulant Use Post-stroke?

The primary rationale for utilizing anticoagulants post-stroke involves the prevention of subsequent ischemic events by mitigating thromboembolic risks, notably in individuals diagnosed with atrial fibrillation (AF), while concurrently minimizing the potential for HT [13-16]. In this patient demographic, strokes are frequently more extensive and exhibit a higher susceptibility to bleeding complications; thus, a careful equilibrium must be achieved between preventing recurrent ischemic episodes and effectively managing the risk of hemorrhagic conversion. Anticoagulation serves to curtail the formation of blood clots within the heart, thereby diminishing the likelihood of recurrent ischemic incidents and subsequent mortality. The restoration of brain perfusion in the initial hours following a stroke stands out as a crucial strategy for mitigating brain damage.

How Does Early Initiation Theoretically Reduce Recurrent Events or Complications?

Theoretically, initiating anticoagulation early may reduce the incidence of subsequent ischemic events and facilitate the management of hemorrhagic risks within the initial two weeks following a stroke [17]. Specifically, commencing OACs early curtails the window of vulnerability to recurrent ischemic events. Clinical observations indicate that the absence of intracranial hemorrhage within 90 days suggests that early initiation does not elevate the risk of hemorrhagic complications [18]. Although early anticoagulation has the potential to prevent recurrent infarctions, it concurrently carries the risk of provoking HT [14]. Conversely, delaying anticoagulation may mitigate the risk of HT but increases the likelihood of secondary stroke [14]. Compared to warfarin, early initiation of non-vitamin K antagonist oral anticoagulants (NOACs) is associated with a more rapid and predictable onset of action and may present a reduced risk of intracerebral hemorrhage (ICH) [19]. However, the early use of NOACs may also increase the risk of hemorrhage, necessitating a careful evaluation of potential benefits against risks [19].

## Review

Early initiation

How Is the Concept of Early Initiation Operationalized Across Various Studies?

Early initiation of anticoagulation following cardioembolic stroke is variably defined, ranging from 0-3 days to 4-14 days post-event [20]. While some protocols suggest initiation within 48 hours for minor to moderate strokes and six to seven days for major strokes [21], a universally accepted definition is lacking. Studies indicate a mean time to anticoagulation initiation of approximately 13 days for reperfusion-treated patients and 12 days for those without reperfusion [22].

Early initiation is associated with a low incidence of thrombotic/hemorrhagic events, a low rate of major hemorrhage, and a low mortality rate, contributing to a high rate of functional independence [23]. In patients undergoing reperfusion therapy, stroke recurrence is observed in 4%, compared to 5% in untreated patients. Major bleeding occurs in 2% of reperfusion-treated patients and 4% of untreated patients. The composite rate of recurrence and major bleeding is 7% in reperfusion-treated patients and 9% in untreated patients [22]. Assessments include radiological HT, symptomatic HT, recurrent ischemic stroke, systemic hemorrhagic complications, National Institutes of Health Stroke Scale (NIHSS) scores at days 7 and 90, modified Rankin Scale (mRS) scores at days 7 and 90, mortality within 90 days, and quality of life assessments at day 90 [24]. Such outcomes suggest that earlier intervention decreases the risk of recurrent stroke and results in improved patient outcomes [25]. However, it's important to note that reperfusion after stroke can paradoxically exacerbate tissue damage and inflammation through reperfusion injury, presenting clinicians with a significant challenge [26]. To mitigate the effects of stroke, the European Stroke Organisation guidelines emphasize timely intervention, recommending intravenous thrombolysis with alteplase within 4.5 hours of symptom onset [27]. Furthermore, these guidelines also suggest mechanical thrombectomy up to six hours, or in select cases up to 24 hours, from the time the patient was last known to be well [28]. The American Heart Association or American Stroke Association also updated guidelines to reduce the time from when the patient arrives at the hospital to when they are treated [29].

Which Specific Subpopulations of Patients Derive the Most Pronounced Benefits From the Early Commencement of Anticoagulation Therapy?

The early commencement of direct oral anticoagulants (DOACs) is demonstrably advantageous for secondary stroke prevention in individuals with AF [17]. Patients experiencing ischemic stroke or transient ischemic attack (TIA) secondary to non-valvular AF benefit from the prompt initiation of DOACs, which mitigates the elevated risk of recurrent IS prior to anticoagulation [30]. Similarly, early OAC initiation is beneficial for patients with AF who have experienced an AIS [18]. The early introduction of anticoagulation does not seem to jeopardize patient safety in cases of TIA or small to medium-sized AIS resulting from AF [14]. Furthermore, patients presenting with covert brain infarcts, AF, and first-time ischemic stroke benefit from early DOAC initiation [21]. Consequently, early anticoagulation proves beneficial for patients with non-valvular AF and AIS, particularly those with TIA or small to medium-sized AIS [31]. In particular, early rivaroxaban initiation is especially beneficial for individuals with nonvalvular AF who have experienced minor strokes or TIAs, especially those with small or medium-sized infarctions, as starting treatment within 14 days of the index event is associated with reduced rates of ischemic stroke recurrence and major bleeding [32]. However, it is important to note that studies indicate that changing the type of anticoagulant after the index event was not associated with a decreased risk of ischemic stroke recurrence [33]. While advancements in AIS management have increased access to treatment via thrombolysis and thrombectomy, early detection remains critical [34]. The aging population is expected to drive increases in post-stroke patients, making effective anticoagulant strategies essential [35].

Delayed initiation

What Constitutes a Delayed Start in the Context of Anticoagulation Therapy Following AIS?

Delayed initiation is variably defined as starting between 7 and 14 days post-stroke [36], initiating DOACs beyond seven days after the index event [15], or initiating NOACs more than 14 days after the acute stroke [37]. In the context of minor strokes, delayed initiation is considered to be day 3-4, day 6-7 for moderate strokes, and day 12-14 for major strokes [38].

What Clinical Outcomes Have Been Documented in Studies Evaluating Delayed Anticoagulation Initiation Strategies Post-AIS?

Within the first three months post-stroke, thrombotic or hemorrhagic events have been observed in 2.2% of patients, comprising 13 embolic/ischemic events and seven hemorrhagic events. Functional independence was noted in 68.5% of patients at the three-month mark, with a 3% mortality rate attributed to various causes, including the index cerebral infarction, intracranial hemorrhage, pneumonia, pulmonary embolism, and other factors [23]. Assessments encompassed symptomatic intracranial hemorrhage, major extracranial bleeding, recurrent ischemic stroke, systemic embolism, vascular death, functional status at 90 days, myocardial infarction at 90 days, TIA and undetermined stroke at 30 and 90 days, major cardiovascular events at 90 days, NIHSS at 90 days, and silent brain lesions at 90 days [38]. The daily recurrence risk ranges from 0.4% to 1.3%, with an elevated risk of symptomatic ICH associated with early anticoagulation using vitamin K antagonists (VKAs) or heparins. Primary outcomes include recurrent ischemic stroke, symptomatic ICH, and systemic arterial embolism, while secondary outcomes encompass all-cause and cardiovascular mortality, recurrent ischemic stroke, systemic embolism, venous thromboembolism, symptomatic ICH, major extracranial bleeding, and clinically relevant non-major bleeding [39]. Delayed initiation is correlated with an increased risk of recurrent embolic events within the initial 30 days, alongside higher rates of symptomatic intracranial hemorrhage and recurrent AIS, thus underscoring concerns regarding HT risk [31]. The composite rate of recurrence and major bleeding is 7% in reperfusion-treated patients and 9% in untreated patients at 90 days. Early recurrence manifests in 4% of reperfusion-treated patients and 5% in untreated patients, whereas major bleeding occurs in 2% of reperfusion-treated patients and 4% in untreated patients [22].

Are There Specific Patient Groups or Clinical Scenarios Where a Delayed Initiation of Anticoagulation Is Deemed More Appropriate?

A delayed initiation of DOACs is potentially advisable for individuals who have experienced parenchymal hemorrhage, as early commencement in these cases may lead to poorer functional outcomes [40]. Current guidelines suggest delaying anticoagulation for 12 to 14 days following a major stroke [41]. Additional factors influencing the decision to delay DOAC initiation encompass high stroke severity, large infarct size, advanced age, elevated diastolic blood pressure, a higher Congestive Heart Failure, Hypertension, Age, Diabetes, Stroke/Transient Ischemic Attack-Vascular Disease, Sex (CHA_2_DS_2_-VASc) score, a history of or predisposition to bleeding [23], spontaneous parenchymal hemorrhage grades PH1 or PH2, recent history or clinical presentation indicative of ICH, subarachnoid hemorrhage (SAH), arteriovenous malformation (AVM), aneurysm, or cerebral neoplasm [24], uncontrolled hypertension, bleeding disorders, primary intracranial hemorrhage, HT of ischemic stroke, or the presence of cerebral microbleeds (CMBs) within or adjacent to the infarct, or more than three CMBs in the brain [31].

Comparative outcomes: early versus delayed initiation

How Do the Rates of Recurrent Stroke, Systemic Embolism, Major Bleeding, and Mortality Differ Between Early and Delayed Initiation in Comparative Studies?

The incidence of recurrent stroke, systemic embolism, major bleeding, and mortality did not demonstrate significant differences between early and delayed anticoagulation initiation [20]. Specifically, early initiation was associated with a 1.7% rate of recurrent stroke, while late initiation showed a rate of 1.2%; intracranial hemorrhage rates were 0.1% and 0.3% for early and late initiation, respectively [30]. When comparing apixaban to warfarin, disparities emerged in recurrent stroke/TIA, mortality, symptomatic hemorrhages, and the primary composite outcome [14]. It is noteworthy that the rates of recurrent stroke and major bleeding fluctuate considerably based on the timing of initiation: 12.4% with initiation within two days, 2.1% with initiation between three and 14 days, and 9.1% with initiation beyond 14 days post-acute stroke [37]. Perioperative management strategies advocate for holding DOACs one to two days before surgery, depending on bleeding risk and resuming them one to three days post-surgery [42]. Initiation of anticoagulation should be avoided for at least eight hours after surgery, and hemostasis must be ensured before commencing such therapy [43]. Balancing the risks of ischemia and bleeding is crucial in antithrombotic treatment following acute coronary syndromes [44]. The comparative benefits and risks of early versus delayed initiation are summarized in Table 1.

**Table 1:** Comparative Benefits and Risks of Early Versus Delayed Oral Anticoagulant Initiation After Acute Ischemic Stroke Adapted and synthesized from published studies [17,18,23,30,45]. Table credits: Authors

Strategy	Potential Benefits	Potential Risks
Early Initiation	May decrease the occurrence of ischemic events; may be justifiable due to the elevated risk of recurrent ischemic stroke before DOAC initiation; fewer recurrent strokes and systemic embolisms; generally regarded as safe and practical.	Potential increase in bleeding risk, although research indicates this risk is minimal; could elevate the likelihood of recurrent ischemic lesions.
Delayed Initiation	May decrease hemorrhagic events.	Might correlate with a greater risk of intracranial hemorrhage.

What Are the Primary Risks and Benefits Associated With Each Strategy?

Initiating anticoagulation early may decrease the occurrence of ischemic events, whereas delayed initiation may decrease hemorrhagic events [17]. Early initiation does not seem to elevate the risk of HT compared to delayed initiation; initiating early may be justifiable due to the elevated risk of recurrent ischemic stroke before DOAC initiation [30]. The main concern with early initiation involves a potential increase in bleeding risk, although research indicates this risk is minimal; the main advantage is the potential for fewer recurrent strokes and systemic embolisms [23]. Early initiation could elevate the likelihood of recurrent ischemic lesions but is generally regarded as safe and practical, while delayed initiation might correlate with a greater risk of intracranial hemorrhage [45]. A consolidated summary of these outcomes is presented in Table 2.

**Table 2:** Summary of Findings Comparing Early Versus Late Oral Anticoagulant Initiation After Acute Ischemic Stroke ^†^ Estimates derived from pooled cohort/registry data; values are approximate and not direct RCT event rates. OAC: oral anticoagulant; RCT: randomized controlled trial Data synthesized from cohort and registry studies [16,18,30,37]. Table credits: Authors

Outcome	Early OAC (Absolute Risk)	Late OAC (Absolute Risk)	Absolute Risk Difference	Interpretation
Recurrent ischaemic stroke (≤ 90 d)	≈ 18 per 1,000 patients^†^	≈ 12 per 1,000 patients	+ 6/1 000 (↑)	Small, non-significant excess of early recurrence
Symptomatic intracranial haemorrhage (sICH)	≈ 3 per 1,000	≈ 4 per 1,000	- 1/1 000 (↓)	Early start does not increase sICH; point estimates slightly favour early timing
All-cause mortality (30-90 d)	≈ 47 per 1,000	≈ 57 per 1,000	- 10/1 000 (↓)	Trend toward lower mortality with early OAC

Potential benefits of early use

Warfarin

Early administration of warfarin may offer advantages, such as decreasing the likelihood of recurrent ischemic incidents and demonstrating safety, as evidenced by the absence of ICH within 90 days, as well as its efficacy in the secondary prevention of stroke for individuals with AF [18]. The correlation between early initiation and reduced recurrence suggests that commencing treatment as early as possible is advantageous.

Dabigatran

Early initiation of dabigatran is potentially advantageous due to its lack of association with increased HT risk in brain infarcts and its potential to mitigate recurrent ischemic stroke risk, considering that a substantial proportion of such strokes occur before DOAC initiation [30]. Evidence suggests that early commencement correlates with decreased recurrence rates, indicating potential benefits such as reduced ischemic events, a favorable safety profile with no observed ICH within 90 days, and effectiveness in secondary stroke prevention among individuals with AF [18]. Further advantages include preventing early ischemic stroke recurrence, ensuring the safety and practicality of early initiation, and reducing symptomatic HT, as well as stabilizing asymptomatic petechial HT [46]. Moreover, research indicates that early dabigatran use is linked to a low risk of intracranial hemorrhage and no significant increase in recurrent ischemic events when compared to delayed initiation [15], alongside a swifter and more predictable mechanism of action than warfarin, and a potentially diminished risk of ICH [19].

Apixaban

Initiating apixaban treatment early may yield several benefits, such as a reduced incidence of recurrent ischemic events, as indicated by the absence of such events in the early initiation group [17]. Furthermore, early apixaban administration does not appear to increase the risk of HT of brain infarcts and may potentially decrease the risk of recurrent ischemic stroke, considering that many recurrent strokes occur before DOAC initiation [30]. Additional advantages of early apixaban initiation include a decreased risk of recurrent ischemic events, a favorable safety profile with no ICH within 90 days, and increased effectiveness in secondary stroke prevention for patients with AF [18]. This is further substantiated by evidence suggesting that early initiation is associated with a decreased risk of recurrence, implying that commencing OACs as soon as clinically appropriate may be advantageous [18]. Moreover, early apixaban usage is linked to the prevention of recurrent infarctions and lower rates of recurrent strokes/TIA, mortality, fatal strokes, and symptomatic hemorrhages, without compromising patient safety [14]. Evidence also indicates that early apixaban usage is associated with numerically lower rates of ischemic stroke and mortality, along with the absence of symptomatic ICHs, suggesting its potential safety and efficacy in acute secondary stroke prevention for eligible patients [16]. Furthermore, early initiation of apixaban may reduce the risk of recurrent ischemic stroke, symptomatic intracranial hemorrhage, major extracranial bleeding, systemic embolism, or vascular death within 30 days, as evidenced by lower numerical rates in the early DOAC-initiated group compared to the late group [47]. Finally, apixaban provides a faster and more predictable onset of action compared to warfarin, along with a decreased risk of ICH and the potential for more immediate protection against recurrent ischemic strokes [19].

Rivaroxaban

Early initiation of rivaroxaban may offer benefits, including no increased risk of HT in brain infarcts and a potential reduction in recurrent ischemic stroke risk, given that a significant number of these strokes occur before DOAC initiation [30]. Early rivaroxaban use may also decrease the risk of recurrent ischemic events and is considered safe, with no observed ICH within 90 days, and effective for secondary stroke prevention in individuals with AF [18]. Although not specific to rivaroxaban, research suggests that early DOAC use is associated with a low risk of intracranial hemorrhage and no significant increase in recurrent ischemic events compared to delayed initiation [15]. Additional advantages of early rivaroxaban administration include numerically lower rates of ischemic stroke and mortality, along with the absence of symptomatic ICHs, indicating its potential safety for acute secondary stroke prevention in eligible patients [16]. Furthermore, rivaroxaban has a more rapid and predictable onset of action compared to warfarin, and a potentially reduced risk of ICH [19].

Edoxaban

Initiating edoxaban treatment early may offer several advantages, including no increased risk of HT of brain infarcts and a potential reduction in the risk of recurrent ischemic stroke, especially considering that a significant proportion of recurrent ischemic strokes occur before DOAC initiation [30]. Early edoxaban use is also associated with a decreased risk of recurrent ischemic events, a favorable safety profile characterized by the absence of ICH within 90 days, and efficacy in the secondary prevention of stroke among patients with AF [18]. Furthermore, studies suggest that early initiation correlates with a reduced risk of recurrence, indicating the potential benefits of commencing edoxaban as soon as possible [18]. Additional benefits of early DOAC use include numerically lower rates of recurrent ischemic stroke, symptomatic intracranial hemorrhage, major extracranial bleeding, systemic embolism, or vascular death within 30 days, suggesting both safety and efficacy [47]. Moreover, early edoxaban use offers a more rapid and predictable onset of action and a lower risk of ICH compared to warfarin, potentially leading to greater efficacy and safety in patients with AIS and AF [19].

Potential risks of late use

Warfarin

The delayed administration of warfarin carries several potential risks, including an elevated incidence of acute recurrent ischemic events [18] and an augmented risk of subsequent stroke [14]. Furthermore, the late initiation of warfarin is correlated with an increased risk of ICH in comparison to NOACs, a phenomenon attributed to warfarin's delayed onset of therapeutic action [19].

Dabigatran

The delayed commencement of dabigatran therapy is associated with a greater incidence of acute recurrent ischemic events, wherein the risk of recurrence escalates with each day of postponement [18]. Furthermore, deferred initiation of dabigatran correlates with increased rates of both ischemic stroke and all-cause mortality relative to early intervention [16]. However, the temporal aspect of dabigatran administration does not appear to exert a substantial influence on the incidence of symptomatic ICH [16].

Apixaban

The late use of apixaban may increase the risk of recurrent embolic events, as early initiation of DOACs appears to be more advantageous than delaying treatment beyond the initial two weeks post-stroke [17]. Furthermore, the delayed administration of apixaban is linked to a greater incidence of acute recurrent ischemic events, which correlates with elevated severity and risk scores, suggesting that postponing initiation increases the risk of recurrence [18]. However, the risk of ICH within 90 days does not show a corresponding increase [18]. In addition, delaying apixaban use is associated with a higher risk of secondary stroke [14] and increased rates of ischemic stroke and all-cause mortality when compared to early initiation [16]. Notably, there is no increased risk of symptomatic ICH associated with the late use of apixaban [16].

Rivaroxaban

Delayed administration of rivaroxaban is associated with increased rates of ischemic stroke and all-cause mortality compared to early initiation. However, the risk of symptomatic ICH is not increased with delayed initiation [16].

Edoxaban

The late administration of edoxaban is associated with a higher incidence of acute recurrent ischemic events [18]. Given that the delayed initiation of OACs correlates with an increased risk of recurrence, early commencement of edoxaban is crucial to mitigate this risk. Furthermore, deferring edoxaban treatment is associated with elevated rates of ischemic stroke and all-cause mortality compared to early initiation [16]. However, delayed edoxaban initiation does not appear to increase the risk of symptomatic ICH [16]. A side-by-side overview of anticoagulants is shown in Table 3.

**Table 3:** Comparative Benefits and Risks of Early Versus Delayed Oral Anticoagulant Initiation by Agent TIA: transient ischemic attack; ICH: intracerebral hemorrhage; INR: international normalized ratio Evidence derived from multiple trials and registries [14-19,23,30,32,37,45,47]. Table credits: Authors

Anticoagulant	Benefits of Early Initiation (≤14 Days)	Risks of Late Initiation (>14 Days)
Warfarin	Cuts recurrent ischemic stroke/TIA risk; effective in secondary prevention; no excess ICH within 90 days in early cohorts; clear relation between timing and recurrence	Higher incidence of recurrent stroke/TIA when delayed; greater overall secondary-stroke risk; higher ICH risk until INR is therapeutic
Dabigatran	Reduces recurrent stroke risk if started early; safe in acute settings; may reduce symptomatic ICH; rapid onset and lower ICH risk than warfarin	More recurrent ischemic events with delay; higher ischemic stroke and mortality rates if initiation is postponed
Apixaban	Early use lowers recurrent stroke/TIA and composite vascular outcomes; maintains safety; rapid onset; lower ICH risk vs warfarin	Higher recurrence if initiation is delayed beyond two weeks; increased risk with higher baseline stroke severity; higher ischemic-stroke and mortality rates with late start
Rivaroxaban	Early use reduces recurrent strokes/deaths; no excess ICH within 90 days; effective secondary prevention; faster onset than warfarin	Higher recurrent ischemic events and mortality when delayed; symptomatic ICH risk unchanged
Edoxaban	No increased hemorrhagic transformation; safe with early use; may cut recurrence	Higher recurrence when initiation is postponed

Is There Evidence From RCTs or Observational Studies That Definitively Supports One Timing Over Another?

The START trial suggests the potential superiority of early initiation over delayed initiation; however, definitive support for either timing strategy remains inconclusive [17]. In addition, an RCT has indicated the non-inferiority of early NOAC initiation compared to delayed initiation [45]. To provide more conclusive results, ongoing trials are in progress. Therefore, early initiation is considered safe and should be taken into account for acute secondary stroke prevention [16]. Moreover, this study furnishes RCT evidence suggesting that early initiation may offer advantages in diminishing recurrent ischemic stroke, though the uncertainty is suggested by the confidence intervals [2]. Furthermore, an RCT suggests that earlier initiation might be more beneficial than delayed initiation within the initial two weeks [48].

Special clinical considerations

How Should the Presence of Large Infarcts or HT Influence Timing Decisions?

The timing of OAC initiation following a stroke is determined in part by the severity and presence of large infarcts or HT [18]. These factors are crucial because early anticoagulation could exacerbate HT [14], and early DOAC initiation might worsen outcomes for individuals with parenchymal hemorrhage [40], whereas delaying anticoagulation elevates the potential for secondary stroke [14]. Consequently, clinicians might postpone anticoagulation due to concerns about intracranial hemorrhage [36]. Furthermore, certain study protocols exclude patients with specific grades of hemorrhage, highlighting a significant risk of further bleeding [24]. However, the presence of substantial infarcts or HT does not inherently amplify the risk associated with early DOAC administration compared to delayed initiation [30].

What Specific Recommendations Exist for Initiating Anticoagulants in the Presence of Thrombolysis or Thrombectomy?

In cases where thrombolysis or thrombectomy has been performed, guidelines suggest the use of a 24-hour post-treatment CT scan to establish a baseline before initiating anticoagulation, and repeat imaging should be conducted if rapid clinical improvement is observed [38,24]. Consequently, the decision regarding the timing of anticoagulation should take into account the presence of thrombolysis or thrombectomy [22].

How Do Patient Factors Influence Timing Considerations?

Individualized factors, such as advanced age, impaired renal function, and the presence of mechanical heart valves, significantly affect the determination of when to start anticoagulation [31]. Clinical trials often exclude these criteria, which implies that tailored strategies for anticoagulation timing may be needed for certain conditions [38]. Moreover, factors like renal dysfunction and low body weight may necessitate dosage adjustments, emphasizing their importance in deciding the best time for anticoagulant therapy [24].

## Conclusions

Current data support starting DOACs soon after an ischaemic stroke, but the exact timing must be tailored to stroke severity and imaging findings. For most patients with minor to moderate infarcts, initiating therapy within 48 hours to five days reduces the risk of early recurrent stroke (≈18 per 1,000 patients vs. 12 per 1,000 with delayed initiation) without a meaningful increase in symptomatic intracranial hemorrhage (≈3 vs. 4 per 1,000). Early initiation also trends toward lower 90-day mortality (≈47 vs. 57 per 1,000). In contrast, large infarcts, haemorrhagic transformation, or recent surgery warrant a more cautious approach, often delaying treatment until day 6 or later once repeat imaging confirms stability. Rather than a prescriptive rule, the commonly cited "2-5-7" framework originates from registry studies and expert consensus and should be viewed as an evidence-informed heuristic, not a formal guideline, pending confirmation from larger randomized trials. Larger RCTs are needed to confirm safety across diverse populations, especially high-risk groups. Long-term outcomes and quality of life remain insufficiently studied, leaving uncertainty about the sustained impact of early versus delayed initiation. As a narrative review, these findings are qualitative and should be considered hypothesis-generating rather than prescriptive guidance.
